# Investigation of prevention of mother to child HIV transmission program from 2011 to 2017 in Suzhou, China

**DOI:** 10.1038/s41598-018-36623-6

**Published:** 2018-12-24

**Authors:** Tian Gong, Huiying Wang, Xiuyu He, Juning Liu, Qianlan Wu, Jing Wang

**Affiliations:** Suzhou Maternal and Child Healthcare Center, Suzhou Municipal Hospital, the Affiliated Suzhou Hospital of Nanjing Medical University, Suzhou, China

## Abstract

The vertical transmission of HIV, from mother to child remains one of the biggest challenges all over the world. This study evaluated the implementation and effectiveness of the prevention of mother to child HIV transmission (PMTCT) program from 2011 to 2017 in Suzhou. A total of 107 HIV positive women were enrolled in the program, of which 11 were diagnosed through premarital examination, and 96 women were diagnosed through prenatal examination. Among the 96 pregnant women, 67 gave birth to 68 live neonates. Only one infant was diagnosed HIV infected because the HIV positive mother did not enter the PMTCT program until delivery. The HIV prevalence in Suzhou city showed a low-level tendency. To increase the prenatal health utility and antiretroviral medication compliance of the migrant population in Suzhou, there are improvements to make in order to achieve the 90-90-90 targets.

## Introduction

The high mortality rate and rapid transmission of human immunodeficiency virus (HIV) make it a worldwide public health problem, that is mostly spreading in the countries with middle and low financial resources^1–3^. There were approximate 36.7 million people living with HIV in 2016, and about 2.1 million of those were less than 15 years old^4^. As a result of the scale-up of antiretroviral treatment, there is a downward trend of new HIV infections, especially in eastern and southern Africa^5^. It was estimated that the number of children newly infected with HIV throughout the world declined by 47% from 2010 to 2016, as the rate of HIV positive pregnant women using antiretroviral therapy to prevent vertical transmission to their children rose from 47% to 76% during the same period^6^.

The vertical transmission of HIV, which is the transmission of HIV from mothers to their children during pregnancy, delivery or breastfeeding, remains one of the most challenging public health problems all over the world^7^. The use of antiretroviral therapy during pregnancy and breastfeeding is necessary for the prevention of mother-to-child transmission of HIV. There is evidence showing that starting antiretroviral therapy as early as possible has multiple benefits including protecting people living with HIV from AIDS-related illness and preventing HIV transmission^6^. The mother-to-child transmission of HIV (MTCT) is the most common mode of transmission of HIV to children. The proportion of children newly infected with HIV through MTCT is over 90%. The interventions aiming to prevent vertical transmission of HIV, improving life quality of HIV infected mothers and their children and protecting both mothers and their children from AIDS-related illness are known as prevention of mother to child transmission of HIV (PMTCT)^8,9^.

According to the implementation plan of the PMTCT program in China, every pregnant woman who receives prenatal care for the first time or gives birth at a hospital, should receive HIV counseling and testing. When a pregnant woman is diagnosed as HIV positive, she will be referred to a specialized hospital for future examination and medication. Free antiretroviral medications are provided to HIV positive women regardless of their CD4 + T lymphocyte count and viral load. These relevant tests should be carried out before and during the antiretroviral treatment, after which the therapeutic effects and HIV infection status are assessed in combination with the clinical symptoms.

Free antiretroviral medication should be provided to the infant as soon as possible (within 6 to 12 hours) after birth Early diagnosis and HIV detection are performed at the 6^th^ week and 3^rd^ month after birth. Regular follow-ups and physical examinations of the infants are performed at the 1^st^, 3^rd^, 6^th^, 9^th^, 12^th^ and 18^th^ month after birth to determine whether there are infection symptoms. For exposed children who have not received early diagnostic tests, HIV antibody tests and if necessary supplementary tests should be carried out at the 12^th^ and 18^th^ month after birth to identify HIV infection.

PMTCT is one of the greatest public health successes of the past 20 years. The rate of MTCT has been reduced to 1% or less when comprehensive prophylactic strategies are implemented^10^. The prevention measures include providing antiretroviral therapy for HIV pregnant women during pregnancy, delivery, and postpartum, providing antiretroviral medications to the infants, elective cesarean section delivery, advocating formula feeding and avoiding breast feeding. For women for whom breastfeeding is the only feeding option, taking antiretroviral medications during the whole period of breastfeeding is recommended by the World Health Organization (WHO)^10,11^.

In the absence of intervention programs to prevent mother-to-child transmission of HIV, the risk of HIV transmission in the uterus and during childbirth is 15–30%, and the risk increases to 20–45% in the case of breastfeeding^7^. It has been reported that the worldwide expanded access to PMTCT services can decrease the risk of vertical transmission of HIV to less than 2%, however, there has been less success in low-income countries due to a variety of factors such as insufficient knowledge of HIV of individuals and limited economic resources^7,12,13^.

Generally, the national HIV epidemic remains at a low-prevalence in China, but clusters of high prevalence exist in some areas of Yunnan, Xinjiang and Henan^14,15^. Despite the Chinese government’s enormous efforts toward HIV/AIDS prevention and control, the HIV epidemic continues to grow. Around the country, an estimated 4.37 per 100,000 people were living with HIV, and 3.21 per 100,000 people were identified as AIDS patients^16^. By the end of 2017, 2.39 per 100,000 people died of AIDS-related illness. HIV prevention has become one of the most severe challenges for national health and development.

Suzhou city is a national historical and cultural city located in the southeast of Jiangsu Province. Nowadays Suzhou is one of the most important central cities of the Yangtze River Delta and famous for the national High-tech industrial base. Suzhou has a population of 10,684 million residents of which almost half are migrants. The economic development in Suzhou has attracted a large migrant population, which includes both top talents and low-income workers. In 2017, the total number of pregnant women and live births in Suzhou were 134,252 and 135,471 respectively, ranking first in Jiangsu province.

In Jiangsu province, 1317 new HIV/AIDS cases were reported in 2017, and 199 patients died of AIDS. The incidence of HIV /AIDS was 0.165‰ in Jiangsu. In Suzhou 692 new HIV/AIDS cases were reported in 2017, a decrease of 4.0% compared to the same period the previous year. Among the newly reported cases, male patients decreased by 7.0%, and female patients increased by 26.2% over the same period. The main transmission route was sexual transmission, including 59.1% male to male transmission and 40.3% heterosexual transmission. From 2012 to 2017, the newly reported male HIV/AIDS cases increased from 341 to 610, and the newly reported female HIV/AIDS cases increased from 34 to 82. The treatment rate increased from 58.1% to 90.1% and the mortality decreased from 3.9% to 1.4%.

The program of prevention of mother-to-child transmission of HIV/AIDS, syphilis, and hepatitis B is an important part of maternal and child health projects. The program is an important measure to protect women and children from AIDS, syphilis, and hepatitis B infection. According to the requirements of the national and provincial major maternal and child health project, Suzhou launched the PMTCT program in 2011, and integrated it with routine maternal and child health care. There are ten districts in Suzhou city, the maternal and child healthcare institution in every district is responsible for reporting information on HIV positive pregnant women and the follow-up of HIV positive pregnant women and their children. If a pregnant woman is diagnosed as HIV positive, the medical staff at the maternal and child healthcare institutions will advise the patients to be referred to a specialized hospital for further diagnosis and treatment. All the HIV positive pregnant women give birth in this specialized hospital. These measures are implemented to improve the compliance of maternal antiretroviral medication use.

After years of actively exploring suitable management mode for preventing mother to child transmission of AIDS, syphilis and hepatitis B in Suzhou, the program has made some progress.

## Results

From the year 2011 to 2017, a total of 96 pregnant women were diagnosed with HIV during prenatal care examination. Among these 96 HIV positive pregnant women, 14(14.58%) HIV positive women were local residents and 82(85.41%) women were from the migrant population. The majority of the HIV positive women, 76(79.17%), were of the Han ethnic group and other 20(20.83%) were from ethnic minorities. Among the ten districts in the city, Zhangjiagang had the highest rate, reporting 23 HIV positive women during the past seven years. Taicang had the lowest rate with three HIV positive women.

From 2011 to 2017, the number of pregnant women and live births increased from 102,602 to 126,281 and 103,056 to 127,327, respectively (Table 1). During those seven years, a total of 107 HIV positive women were reported by the PMTCT program, of which 96 women were diagnosed through prenatal examination, and 11 women were diagnosed during pre-marital medical examination. The prevalence of HIV positive pregnant women showed an increase in these years, and this increase was statistically significant (*χ2* = 14.16, *p* = 0.028). This increase was mainly due to two causes. One cause was an increased detection rate due to the scaled-up prenatal care examination; another cause was the accumulation of previously infected patients. Of the HIV positive women, 66 were diagnosed during antenatal care, 15 around delivery, and 15 during other procedures such as artificial termination of pregnancy. The number of pregnant women diagnosed during antenatal care was higher than the number of diagnosed at other times, and the difference was statistically significant (*χ2* = 25.65, *p* = 0.012). The number of HIV positive pregnant women using antiretroviral medications was 57 (59.38%), and 52 of these received antiretroviral medications adherent to the program requirements. The rate of HIV positive pregnant women using antiretroviral medications from 2011 to 2017 varied, and the difference was statistically significant (*χ2* = 20.08, *p* = 0.003). The numbers of HIV positive women taking antiretroviral drugs during antenatal, delivery and postpartum also varied, and the difference was statistically significant (antenatal medication use: *χ2* = 19.87, *p* = 0.003; delivery medication use: *χ2* = 15.17, *p* = 0.019; postpartum medication use: *χ2* = 17.52, *p* = 0.008).

**Table 1:** The basic information of pregnant women and HIV positive women from 2011 to 2017.

Year	Number of pregnant women	Number of live births	Number of HIV positive pregnant women		Confirmation time of pregnant women			Use of antiretroviral medication		
			n	Incidence (1/10000)	Antenatal care	Delivery	Others	Antenatal	Delivery	Postpartum
2011	102602	103056	11	1.07	8	3	0	4	4	2
2012	115157	115745	10	0.87	7	2	1	7	7	7
2013	116992	117738	18	1.54	16	2	0	10	10	10
2014	120793	121592	9	0.75	7	2	0	7	7	7
2015	120989	121835	7	0.58	3	2	2	3	3	3
2016	122439	123493	17	1.39	14	0	3	16	14	14
2017	126281	127327	24	1.90	11	4	9	10	9	9
P value			0.028(χ2 = 14.16)		0.012(χ2 = 25.65)			0.003(χ2 = 20.08)		

P < 0.05 was considered as statistical significance.

The average age of the HIV positive pregnant women was 27.99 ± 5.79 years (range:18 to 42 years), and the average age of their sexual partners was 27.03 ± 16.18 years. Most pregnant women (n = 58, 60.42%) and their sexual partners (n = 51, 53.13%) were between 20 to 35 years old (Table 2). The second largest age group of pregnant women (n = 33, 34.38%) and their sexual partners (n = 32, 33.33%) was below 20 years old. Most of the HIV positive women (n = 51, 53.13%) and their sexual partners (n = 32, 33.33%) reported junior high school as highest education (Table 2). According to the records, 54 of the HIV positive women were housewives, while the other women were mainly engaged in low- income work. The HIV positive women showed a tendency of low educational level, low age and low income.

**Table 2:** The educational background and age of HIV positive pregnant women and sexual partners.

	Educational background					Age		
	Primary school and below	Secondary school	High school	Bachelor degree and above	No detailed information	≤20 years old	20~35 years old	35~45 years old
HIV positive women	17	51	16	11	1	33	58	5
Sexual partners	12	32	20	16	16	32	51	13

From the records of the PMTCT reporting system, we know that 69 pregnant women were infected through sexual behavior, either by having sex with an HIV positive partner or by having paid sex, accounting for 71.9% of the transmission route. Three women were infected through intravenous drug use and one through blood transmission. By comparison, 14 partners of the pregnant women were infected through sexual behavior, one through intravenous drug use and one by blood transmission, while of most partners no detailed information was available. Detailed transmission information is shown in Table 3.

**Table 3:** The transmission route of HIV positive pregnant women and sexual partners.

Transmission route of HIV	Number of HIV positive women	Number of sexual partners
Sexual transmission	69(71.9%)	14(14.6%)
Blood transmission	1(1.0%)	2(2.1%)
Drug use	3(3.1%)	1(1.0%)
No detailed information	23(24.0%)	79(82.3%)

Of the 96 pregnant women, 67 pregnant women gave birth, 26 pregnant women underwent artificial termination of pregnancy, two women had a spontaneous abortion, and one was lost to follow up (Table 4). Of the 67 women who gave birth, 57 received selective Caesarean sections, and ten had a vaginal delivery. Of the women who gave birth, 21 women had pregnancy complications, of which preterm birth was the most common. Four HIV positive women were co-infected with syphilis.

**Table 4:** The pregnancy outcomes and complications of HIV positive women.

Pregnancy outcomes				Mode of delivery		Pregnancy complications							
Delivery	artificial termination of pregnancy	spontaneous abortion	Miss to follow up	Caesarean section	Vaginal delivery	FGR	GDM	Preterm birth	PROM	Oligohydramnios	Fetal distress	Syphilis	Breech position
67	26	2	1	57	10	1	1	11	1	1	1	4	1

From the year 2011 to 2017, 67 HIV positive pregnant women gave birth to 68 neonates; only one woman gave birth to twins. There were 35 male neonates and 33 female neonates (Table 5). The average weights of the male and female neonates were 2.96 ± 0.62 Kg and 2.85 ± 0.42 Kg respectively. The average birth weight of the males was significantly higher than of the females (*χ2* = 174.26; *p* < 0.0001). The recommended antiretroviral regimens prescribed to the neonates born to HIV positive women were Zidovudine (AZT) or Nevirapine (NVP). Based on our records, all neonates were given antiretroviral drugs except for one female neonate. This lack of treatment was due to rejection by the family members. According to the follow-ups of the neonates, only one was diagnosed with HIV infection.

**Table 5:** The basic information of neonates.

Gender of neonates	Number of neonates	Average weight(Kg)	Drug use of neonates		
			AZT	NVP	Not using drugs
Male	35	2.96 ± 0.62	16	19	0
female	33	2.85 ± 0.42	12	20	1
Total	68	2.93 ± 0.54	28	39	1

## Discussion

According to this study, from 2011 to 2017, the prevalence of HIV positive pregnant women increased gradually in the Suzhou region. The overall HIV incidence among pregnant women is low. The migrant population and the population with a lower education level and income had a higher risk of being infected. The difficulty in ensuring the effective implementation of the program lies in enhancing the health awareness and medication compliance of the migrant population. Suzhou has a large migrant population, accounting for more than half of the total population. The migrant groups usually do not seek antenatal care actively and regularly, leading to a large part of HIV positive women missing the opportunity for prenatal HIV counseling and testing, and consequently missing the antiretroviral therapy. This increases the risk of mother-to-child transmission of HIV. In the absence of comprehensive prevention measures, the risk of HIV transmission from mother to child could increase to 40%^17^. Providing antiretroviral therapy to pregnant mothers could reduce the risk of infection to lower than 2%, increasing maternal and infant life expectancy^18,19^. According to the PMTCT records, only one infant was diagnosed with HIV infection; this was probably because the HIV infected mother did not receive HIV counseling and testing until delivery. Therefore she did not take antiretroviral medication during pregnancy. Early diagnosis and treatment are the keys to prevent MTCT of HIV.

As a result of scaled-up HIV prevention services, HIV prevalence among children was 47% lower in 2016 than in 2011^6^. HIV positive women of reproductive age are an important source of infection as they can infect both their sexual partners by sexual transmission and their children by vertical transmission. To eliminate the global MTCT of HIV, it is necessary to expand the PMTCT interventions. MTCT of HIV can be almost completely prevented if both the mother and the infant receive ARV treatment as early as possible during pregnancy and after birth. The priority actions recommended by the WHO include preventing HIV infections among women of reproductive age, helping HIV positive women avoid unintended pregnancies, ensuring pregnant women have access to HIV counseling and testing, and providing HIV positive pregnant women with antiretroviral medication to prevent transmission^20^.

Approximately 36.7 million people were living with HIV globally at the end of 2016, among them 1.8 million were newly infected^21^. There is no cure for HIV infection. Effective antiretroviral drugs, however, can control disease progress so that people with HIV can have long and productive lives and can help prevent transmission. Providing antiretroviral therapy for pregnant women is cost-effective. Economic analyse found that investment in HIV treatment services can generate economic returns as a result of increased employment and productivity, and averted future expenses for medical services for both mother and offspring^20,22^. However, gender disparities in education and employment, along with power inequalities between women and men, and the fear of violence may increase HIV vulnerability and limit women’s access to HIV services or adherence to HIV prevention or treatment^22–27^. These make HIV prevention especially difficult for women.

There are two sets of antiretroviral medication regimens recommended for pregnant women in PMTCT program in China, one is the combination of Zidovudine (AZT), Lamivudine (3TC) and Rravavir/ritonavir (LPV/r), and another regime is the combination of tenofovirdisoproxilfumarate (TDF), Lamivudine (3TC) and Efavirenz (EFV). After investigating the antiretroviral treatments of these 96 HIV positive women, only 59.38% of women took the antiretroviral medications; this rate was much lower than the recommended 90% of the program. A total of 39 women did not use antiretroviral medication at all, while five women did not receive standardized antiretroviral treatment of whom two did not take antiretroviral medication during the postpartum period, and three did not take antiretroviral medication both during childbirth and the postpartum period. Even though the antiretroviral medications are free for HIV infected pregnant women, the compliance rate is much lower than expected. While all the HIV infected mothers were referred l to specialized hospitals for HIV diagnosis and were prescribed medications, some HIV infected mothers refused to follow the instructions as they concealed the infection information from family members. Some pregnant women chose to undergo artificial termination of their pregnancy, the medication information of this group of women was not reported in the PMTCT system. Another factor that leads to a low antiretroviral medication rate was that some women did not recognize the importance of antiretroviral medications due to their low educational level.

Among the 68 neonates born to the HIV infected women, only one female neonate did not recieve antiretroviral medication. The overall rate of infants taking antiretroviral medication was 98.53%, which was lower than the recommended 100%. Both the infant and her HIV infected mother both did not take the antiretroviral medication due to the parents’ rejection. The HIV positive women’s knowledge and enrolment are important to guarantee successful PMTCT implementation. What’s more, the support of family members’ is also very important. Most HIV infected women enrolled in the PMTCT program were infected by unprotected sexual behavior. This made them feel embarrassed to talk about their infection status, even with family members or their husbands. The stigma of HIV positive women and the common social perception of HIV causes people at risk of HIV infection to rarely take the initiative to seek HIV counseling and testing in hospitals. The delayed diagnosis of HIV infected pregnant women lead to missed opportunity of taking antiretroviral medication.

In 2014, the Joint United Nations Program on HIV/AIDS (UNAIDS) set the 90–90–90 targets to be reached by 2020. That is by 2020, 90% of people living with HIV will know their status, 90% of those diagnosed will take antiretroviral therapy, and 90% of people on treatment will suppress viral loads^28^. According to the reported data, substantial progress has been made towards the 90–90–90 targets. Globally, more than two thirds of people living with HIV were diagnosed by 2016, 77% of those who knew their HIV status were taking antiretroviral therapy, and 82% of people on treatment achieved viral suppression^6^. Achieving 90-90-90 by 2020 and scaling up to 95-95-95 by 2030 will reduce the number of new HIV infections by90% by 2030^29^.

The Chinese government has made a great effort to achieve the 90–90–90 targets, there is however still a long way to go. Our current research is the first study investigating the epidemiology of HIV in pregnant women in Suzhou. Even though the mother-to-child transmission of HIV was low according to the data presented, there is still room for improvement in the process and details of the program. To diagnose HIV positive pregnant women as soon as possible, it is better for women to visit the hospital seeking antenatal healthcare regularly. However, the healthcare awareness of some migrant groups is poor, and for the previously diagnosed HIV positive pregnant women, the degree of society’s acceptance of HIV and stigma of the patients make some HIV positive pregnant women avoid taking the initiative to seek counseling and testing. This leads to a part of HIV positive pregnant women not being included in the PMTCT program. To increase population awareness of timely diagnosis and antiretroviral medication use against HIV/AIDS, it is necessary to introduce health education among the population rather than avoid talking about it. Some HIV positive pregnant women concealed their HIV infection from their families, this could decrease the compliance of the patients. Based on the patient’s privacy, medical staff could not share the patient’s information without the patient’s consent. However, HIV positive pregnant women are different from the common population, as their health also affects fetal health. This dilemma poses difficulties for medical treatment. Solving this dilemma may depend on future policy adjustments. In addition to investigating the basic information of HIV positive pregnant women, the influence of the mental health of HIV positive pregnant women on medication use, the influence of viral load of the HIV positive pregnant women on fetal health, and other factors should be investigated as well. The PMTCT program and the factors that influence it should be studied further to continue evaluation and improve the program for preventing HIV infection in Suzhou.

## Methods

### Data collection

The data used in this study were obtained from the PMTCT program carried out in hospitals and maternal and child health care centers of Suzhou from 2011 to 2017. From the year of 2011, Suzhou began to implement the PMTCT program and it covered all medical and health care institutions. There were specially trained staffs in charge of this program in these institutions, and they were responsible for collecting and reporting information. The staffs of municipal, county, and district-level maternal and child health care institutions were responsible for summarizing the data and information quality control.

According to the requirements of the maternal health care management system and the PMTCT program in Suzhou, for all women receiving their first prenatal examination or delivering in a hospital, HIV testing and counseling are carried out. For pregnant women with a positive result in an HIV antibody screening test such as the rapid test (RT), enzyme-linked immunosorbent assay (ELISA), chemiluminescence immunoassay (CLIA), or gelatin particle agglutination test (PA), a reexamination would be conducted using the original or another screening test. Samples from women with positive results in the reexamination are further analysed with a supplemental antibody assay or a supplemental nucleic acid assay. Based on the positive results of the supplemental assay, women were diagnosed HIV infection. All methods were performed in accordance with relevant guidelines and regulations of the National Health Commission of the People’s Republic of China.

For HIV positive pregnant women free antiretroviral drugs were provided at the designated hospital, as well as a series of prenatal examinations, and regular follow-ups until delivery. Hospital delivery was provided for all HIV positive pregnant women. The neonates born to HIV positive mothers underwent early blood tests and were given antiretroviral drugs immediately after birth to prevent mother-to-child HIV infection. Early diagnosis and HIV detection were performed at the 6^th^ week and 3^rd^ month after birth. Venous blood of the infant was collected on dried blood spots (DBS) cards; the DBS cards were sent to the regional laboratory center for diagnosis. If the exposed children did not receive early diagnostic tests, HIV antibody and necessary supplemental tests were carried out at the 12^th^ and 18^th^ month to identify HIV infection. Feeding guidance and regular child health care service were provided. The follow-ups and physical examinations were carried out to determine whether there were symptoms of infection at 1, 3, 6, 9, 12 and 18 months after birth. For the children who did not carry out early blood test should receive supplementary tests in 12 and 18 month to identify the status of HIV infection.

### Statistical analysis

The basic information of the HIV positive mothers including marital status, gravidity and parity history, educational background, date of HIV diagnosis, transmission route of HIV, information about the sexual partner, last menstrual period, current pregnancy outcome, and neonatal information were collected. The characteristics of the HIV positive mothers from 2011 to 2017 were analyzed. The statistical difference between the groups was evaluated by the χ2-test or Fisher’s exact test as appropriate. P < 0.05 was considered statistically significant. The analyses were conducted using SPSS 17.0 software (Chicago Illinois, USA).

### Ethical considerations

This study was approved by the Ethics committee of Suzhou Municipal Hospital. The data were collected through routine information questionnaire. All infected mothers were required to complete the questionnaire when they received HIV testing at their first antenatal care or delivery. The informed consent was obtained from the HIV positive mothers. The testing results and antiretroviral treatment information were obtained from medical records. The study was approved by the ethics committee to link the two routinely collected data sets as a municipal PMTCT database. In the final database used in this investigation, only the special numbers for mothers and infants were listed. All personal information was kept confidential.
